# Upper-Limb Robot-Assisted Therapy Based on Visual Error Augmentation in Virtual Reality for Motor Recovery and Kinematics after Chronic Hemiparetic Stroke: A Feasibility Study

**DOI:** 10.3390/healthcare10071186

**Published:** 2022-06-24

**Authors:** Ki-Hun Cho, Mi-Ran Hong, Won-Kyung Song

**Affiliations:** 1 Department of Physical Therapy, Korea National University of Transportation, Jeungpyeong 27909, Korea; mamiya34@gmail.com; 2 Department of Rehabilitative & Assistive Technology, National Rehabilitation Research Institute, National Rehabilitation Center, 58 Samgaksan-ro, Gangbuk-gu, Seoul 01022, Korea; hmiran1004@korea.kr

**Keywords:** error augmentation, robot-assisted therapy, stroke, upper limb, virtual reality

## Abstract

The purpose of this study was to investigate the effect of upper-limb robot-assisted therapy based on visual error augmentation in virtual reality (UL-RAT-VEAVR) for motor recovery and kinematics after chronic hemiparetic stroke. This study applied a single-group pre- and post-intervention study design. A total of 27 stroke survivors (20 males and 7 females; mean age 54.51 years, mean onset duration 12.7 months) volunteered to participate in this study. UL-RAT-VEAVR was performed three times a week for four weeks, amounting to a total of twelve sessions, in which an end-effector-based robotic arm was used with a visual display environment in virtual reality. Each subject performed a total of 480 point-to-point movements toward 3 direction targets (medial, ipsilateral, and contralateral side) in the visual display environment system while holding the handle of the end-effector-based robotic arm. The visual error (distance to the targets on the monitor) in virtual reality was increased by 5% every week based on the subject’s maximum point-to-point reaching trajectory. Upper-limb motor recovery was measured in all subjects using the Fugl–Meyer Assessment (FMA) upper-limb subscale, the Box and Block Test (BBT), and the Action Research Arm Test (ARAT), before and after training. In addition, a kinematic assessment was also performed before and after training and consisted of time, speed, distance, and curvilinear ratio for point-to-point movement. There were significant improvements in both upper-limb motor function and kinematics after 4 weeks of UL-RAT-VEAVR (*p* < 0.05). Our results showed that the UL-RAT-VEAVR may have the potential to be used as one of the upper-limb rehabilitation strategies in chronic stroke survivors. Future studies should investigate the clinical effects of the error-augmentation paradigm using an RCT design.

## 1. Introduction

About 15 million people worldwide experience a stroke every year [1], resulting in various motor impairments, such as limitation of the range of motion, errors in arm movement, and abnormal joint coupling [2]. Motor impairment is one of the most common problems after stroke, and the recovery of upper extremity function is a priority for stroke survivors [3]. Upper extremity motor impairments, which require a high level of fine motor control of the arms and hands, are more likely to result in activity restriction than lower-extremity motor impairments [4]. Although recovery of upper-extremity motor function is essential for stroke patients to take care of themselves and perform activities of daily life (ADLs) [5], only 35–70% of stroke survivors recover functional levels of arm movement, and more than 50% have persistent upper extremity deficits [4,6].

In recent years, there has been a growing trend toward using interactive technology for the restoration of upper-extremity motor control in stroke survivors [7]. Robot-assisted therapy is an advanced technology that is increasingly used in post-stroke upper-extremity rehabilitation [8]. The use of robotics in stroke rehabilitation can provide high convenience, compared with traditional approaches, when performing task-oriented training, and it can provide the advantage of increasing the accuracy of measurement of kinematic results, such as movement speed and trajectory straightness [9]. Generally, upper-limb rehabilitation robots are categorized into the end-effector and exoskeleton types, according to their mechanical structure [10]. The end-effector models are connected to the user at one distal point to allow the reproduction of the dynamic environment that corresponds to ADLs, whereas the exoskeleton models can train specific muscles by controlling joint motion with calculated torque at multiple points [10,11]. In rehabilitation strategies employing the use of robotics, a key factor influencing the recovery of motor control is feedback, which is information provided through an individual’s performance results [4]. The feedback in robotic rehabilitation can be used as a source for providing knowledge about the results of movement performance, and it has been proposed as means to promote motor learning and improve motor performance through two main paradigms—error reduction (ER) and error augmentation (EA) [12].

The ER paradigm, also known as haptic guidance, is based on the hypothesis that, by guiding the subject to the correct movement trajectory, motor learning can be induced through imitation [4,13]. In other words, the ER paradigm aims to reduce a subject’s movement errors during motor performance. In contrast, the EA paradigm uses visual or sensory feedback to magnify the error along the desired trajectory [14]. The ER paradigm is often seen as counterintuitive because it contrasts with conventional approaches that aim to minimize patient movement errors. However, iterative learning of EA has shown the potential to promote movement control [15,16]. In addition, EA learning is considered a major factor in neuroplasticity and the reacquisition of movement skills [16,17]. A previous systematic review [4] suggested that robotic therapy using the EA paradigm is more effective than conventional repetitive practice for upper-extremity motor performance and motor recovery in stroke. In addition, another study reported that EA training showed significant improvement over simple, repetitive practices in upper-extremity motor recovery (Fugl–Meyer Assessment and Wolf Motor Function Test) post-stroke [16]. However, evidence for the effectiveness and therapeutic strategies of EA is still lacking. In other words, it is necessary to standardize therapeutic strategies through more diverse clinical studies [4]. Thus, the purpose of this study was to investigate the effect of upper-limb robot-assisted therapy based on visual error augmentation in virtual reality for motor recovery and kinematics after chronic hemiparetic stroke, providing a feasibility study.

## 2. Materials and Methods

### 2.1. Subjects

This study applied a single-group pre- and post-test study design to investigate the effect of upper-limb robot-assisted therapy based on visual error augmentation in virtual reality for motor recovery and kinematics in chronic hemiparetic stroke survivors. Twenty-seven stroke survivors volunteered to participate in this study. It was approved by the Korea National Rehabilitation Center Institutional Review Board (NRC-2018-02-010) and was conducted in accordance with the approved guidelines. All participants provided informed consent according to the Declaration of Helsinki prior to commencing the study. The inclusion criteria were as follows: (1) at least 6 months after stroke onset; (2) ability to follow the study instructions (≥24 points on the Korean version of the Mini-Mental State Examination); (3) absence of any musculoskeletal condition that could affect the ability to sit safely; and (4) presence of some recovery in the upper extremity (Fugl-Meyer Assessment score 15 to 50) [16]. Exclusion criteria were (1) shoulder subluxation or obvious joint pain of the upper extremity; (2) severe spasticity (modified Ashworth scale < 3); and (3) botulinum toxin injection to the paretic side of the upper extremity within 4 months.

### 2.2. Study Setting 

**Figure 1:**
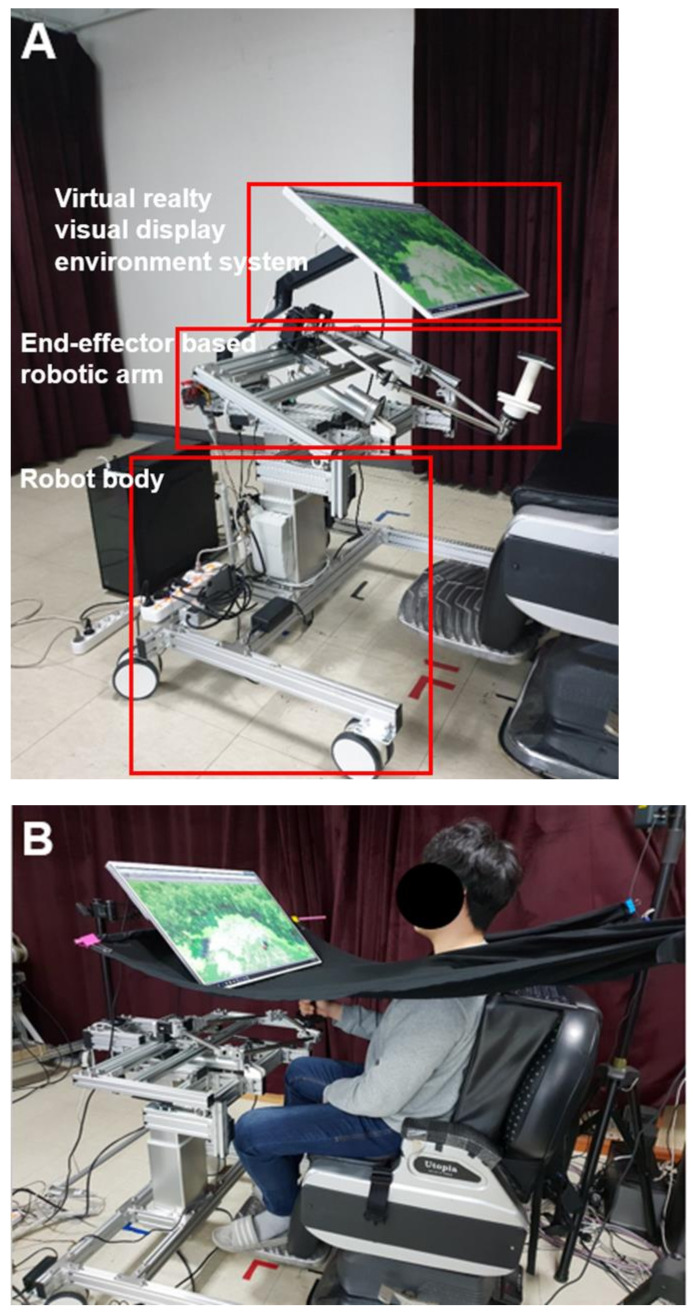
Configuration of NREH (**A**) and setting for upper-limb robot-assisted therapy based on visual error augmentation in virtual reality (**B**).

Upper-limb robot-assisted therapy based on visual error augmentation in virtual reality was conducted with a national rehabilitation center end-effector-based rehabilitation arm at home (NREH), developed at the Korea National Rehabilitation Institute. The NREH is composed of a robot body, an end-effector-based robotic arm, and a visual display environment system in virtual reality (Figure 1A). The robot body is equipped with 4 casters that can be moved and fixed. In addition, the robot body can be adjusted in height (700–1100 mm) through an electric motor. The end-effector-based robotic arm has two degrees of freedom, a five-bar linkage, two torque motors, position encoders, and a handle that the subject can grasp. During upper-limb robot-assisted therapy based on visual error augmentation in virtual reality, the speed, distance, and trajectory of the handle movement were derived from two position encoders. In addition, two torque motors delivered a programmed assistive force to the subject that grasped the handle. An assistance force of 4 N was temporarily provided based on the subjects’ point-to-point movement performance to assist a lack of movement. The visual display environment was provided as virtual reality via a 32-inch monitor (LG Electronics, Seoul, South Korea, model 32QK500). The NREH was designed to adjust the height of the end-effector-based robotic arm and the angle of the visual display environment system, according to the subject’s sitting posture.

### 2.3. Experimental Protocol

Upper-limb robot-assisted therapy based on visual error augmentation in virtual reality was performed three times a week for four weeks, for a total of twelve sessions. Each subject sat in front of the NREH and performed 480 point-to-point movements (160 times in each direction) toward the target in 3 directions (medial, ipsilateral, and contralateral side) while holding the handle of the end-effector-based robotic arm.

In the visual display environment system, the starting point was indicated by a white ball, and the robot’s handle, which was synchronized with the movement of the subject’s arm, was indicated by a red ball. In addition, the target of the point-to-point movement was indicated using a cylindrical barrel. Targets were marked only one at a time, at random, in all three directions, and the subject reached their arm to the marked target, stayed there for a while, and then returned to the starting point. The starting point was remarked at the same location throughout the point-to-point movement. In addition, the red ball that synchronized with the subject’s arm movement was allowed to move in all directions during the point-to-point movement.

**Figure 2:**
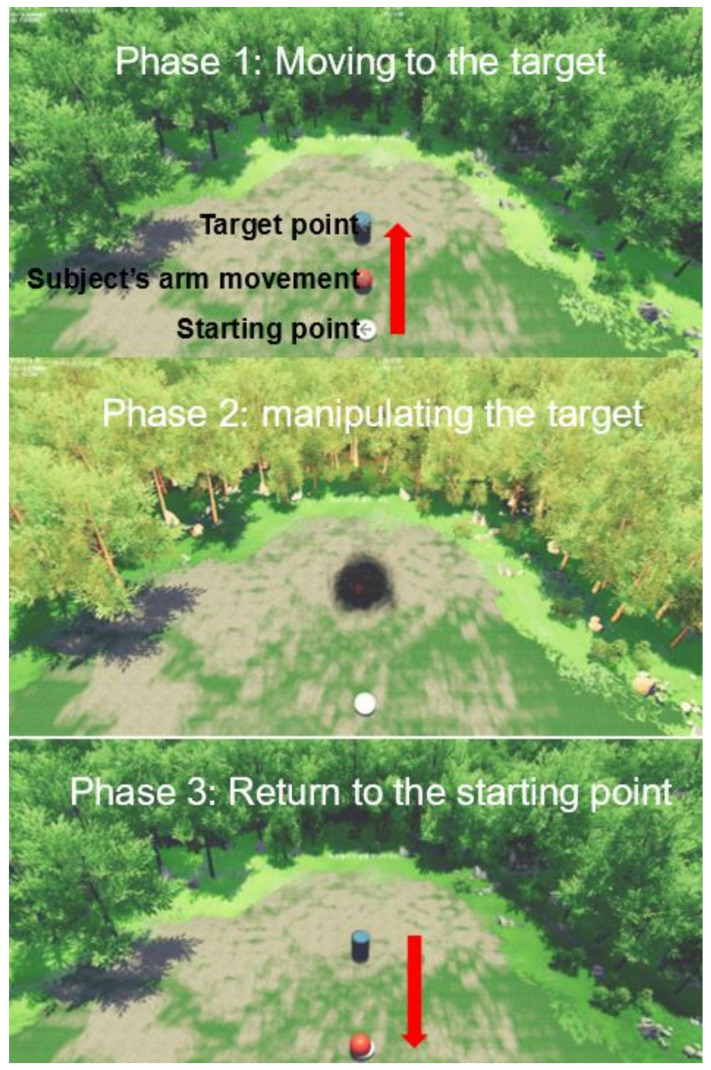
The visual display environment in virtual reality.

The point-to-point movement consisted of three consecutive phases: moving to the target, manipulating the target, and returning to the starting point (Figure 2). In the first phase, the arm (red ball) was moved toward the target (cylindrical barrel) that appeared randomly from the starting position (white ball). In the second phase, when the subject’s arm manipulated the target and stayed for 0.5 s, the target was detonated. Finally, in the last phase, the arm was returned from the target to the starting point. There was no time limit to complete the training, but each session took approximately 40 min (min–max: 35–45 min), including rest time. Due to the difference in rest time, there was a difference in the total training time required for each subject.

Before each training session, the subjects practiced point-to-point movement 10 times in 3 directions, to familiarize themselves with the experimental environment. No visual error augmentation in virtual reality was applied during the practice. After practice, visual error augmentation in virtual reality was applied during the main training session.

The visual error in virtual reality increased by 5% every week based on the subject’s 80% of the maximum point-to-point reaching trajectory (80% of the maximum distance that can be reached in a straight line toward the target) measured before the start of training. In other words, the target position on the monitor did not change during the 4 weeks of training, but at week 2, the subject had to move the arm at 85% of the maximum point-to-point trajectory. In addition, at week 4, the subject had to move the arm at 95% of the maximum point-to-point trajectory. In a pilot test (four stroke survivors) conducted before the start of this study, the visual error increased by 5% every week based on the subject’s 100% of the maximum point-to-point reaching trajectory. However, this challenging task caused malalignment and compensation of the trunk. Therefore, based on several tests and experts’ opinions, 80% of the maximum point-to-point reaching trajectory was set as the reference point for training.

All training sessions were conducted by a licensed occupational therapist, and none of the subjects participated in any other rehabilitation program during the experimental period. In addition, subjects were blocked with a black cloth so that they could not see the robotic arm during training, to enhance the immersion of training (Figure 1B).

### 2.4. Evaluation Procedure

All subjects underwent clinical and kinematic assessments before and after training. Upper-limb motor recovery was assessed using the Fugl–Meyer Assessment (FMA) upper-limb subscale, Box and Block Test (BBT), and Action Research Arm Test (ARAT). On the FMA upper-limb subscale (0–66), higher scores indicate better motor function. The FMA upper-limb subscale was divided into subscores for the proximal unit of the shoulder/elbow (0–42) and the distal unit of the hand/wrist (0–24) [18,19]. The BBT, a quick, simple, and inexpensive test, can be used to measure unilateral gross manual dexterity in a wide range of populations, including stroke patients. The subjects moved as many blocks as possible from one box to the other, using only the hand being tested, for 60 s. A higher number of displaced blocks indicated better gross dexterity [20]. The ARAT is designed to assess upper-extremity performance (coordination, dexterity, and functioning) by measuring four subscales (grasp, grip, pinch, and gross movement). Task performance was graded on a 4-point scale from 0 (unable to complete any part of the hand or arm movement) to 3 (normal performance), with a maximum score of 57 [21].

The kinematic assessment consisted of time, speed, distance, and curvilinear ratio for point-to-point movements. The curvilinearity ratio is the ratio of the straight-line distance between the starting point and the target and was calculated using the following formula:

$$\mathrm{curvilinearity}\;\mathrm{ratio}=\frac{\mathrm{length}\;\mathrm{of}\;a\;\mathrm{straight}\;\mathrm{line}\;\mathrm{from}\;\mathrm{the}\;\mathrm{start}\;\mathrm{point}\;\mathrm{to}\;\mathrm{the}\;\mathrm{target}\;\mathrm{point}}{\mathrm{actual}\;\mathrm{displacement}\;\mathrm{of}\;\mathrm{the}\;\mathrm{hand}}$$

Kinematic data (time, speed, distance, and curvilinear ratio for point-to-point movements) were recorded with the robotic system during the upper-limb robot-assisted therapy based on visual error augmentation in virtual reality. In addition, the recorded kinematic data were calculated using the MATLAB software (MathWorks Inc., Natick, MA, USA) for further analysis.

### 2.5. Statistical Analysis

Data analysis was performed using SPSS (version 21.0; IBM Corp., Armonk, NY, USA). Descriptive statistics and frequency analysis were used to describe the baseline characteristics of the participants. For dependent variable measures, the Wilcoxon signed-rank test was used to compare upper-limb motor recovery and kinematics within groups after visual error augmentation-based robot-arm training. A significance level of 0.05 was used for all tests. A minimum sample size of 27 subjects was calculated using a power calculation tool (G*Power 3.1.9.3 software; Heinrich-Heine University, Düsseldorf, Germany), with power, alpha, and effective size set at 0.80, 0.05, and 0.87, respectively.

## 3. Results

The general characteristics of the 27 stroke survivors (20 males and 7 females; mean age 54.51 years, onset duration 12.7 months) who fulfilled the inclusion criteria are summarized in Table 1. 

Table 2 shows the changes in upper-limb motor recovery (FMA, BBT, and ARAT). Regarding upper-limb motor recovery, there were significant improvements in total FMA units (36.92 to 38.55) and proximal FMA units (25.88 to 27.11), BBT (5.33 to 5.92), and ARAT (16.96 to 18.29) after four weeks of upper-limb robot-assisted therapy based on visual error augmentation in virtual reality (*p* < 0.05). However, no significant improvement was observed in the distal FMA unit.

Table 3 shows the changes in upper-limb kinematics (time, speed, distance, and curvilinearity ratio in three directions: medial, ipsilateral, and contralateral side) for point-to-point movements. There was significant improvement in the three directions of time (ipsilateral: 3.94 to 2.94 s; contralateral: 4.17 to 2.93 s; medial: 3.68 to 3.00 s), speed (ipsilateral: 6.26 to 8.28 mm/s; contralateral: 6.76 to 8.93 mm/s; medial: 7.48 to 9.98 mm/s), distance (ipsilateral: 15.09 to 17.52 mm; contralateral: 17.00 to 19.59 mm; medial: 19.81 to 22.46 mm), and curvilinearity ratio (ipsilateral: 0.61 to 0.71; contralateral: 0.61 to 0.70; medial: 0.61 to 0.71) after 4 weeks of upper-limb robot-assisted therapy based on visual error augmentation in virtual reality (*p* < 0.05).

## 4. Discussion

Our objective in performing this study was to investigate the effect of upper-limb robot-assisted therapy based on visual error augmentation in virtual reality for motor recovery and kinematics in chronic hemiparetic stroke survivors. After four weeks of training, we observed improvements in both upper-limb motor recovery and kinematics of these survivors.

The decrease in upper-extremity motor function due to paralysis is one of the major factors that impair the ability of a person to lead an independent life after a stroke. Therefore, various approaches have been investigated to improve upper-extremity function in stroke patients [22]. Among the various approaches, the field of robotics has recently been increasingly employed to improve extremity motor function after stroke [23]. Compared with conventional approaches, rehabilitation involving robotics is convenient for providing task-oriented training but also has the advantage of ensuring high measurement accuracy of performance metrics, such as trajectory linearity, movement time, speed, distance, and joint range of motion [4,9]. According to the challenge point theory, motor learning is maximized when a task of appropriate difficulty is provided in accordance with the individual skill level of the performer; in that regard, robot arm training is known to be particularly helpful for acute stroke patients with low movement skill levels [24]. In addition, previous research has also shown that robotics can be used to provide more challenging tasks in stroke rehabilitation when combined with various technologies, such as virtual reality and haptics [25]. This has allowed adequate and beneficial training for the more skilled participants [8]. In particular, the combination of robotics with visual error augmentation is suitable for enhancing learning by improving the subject’s concentration while performing simple tasks [26], and for promoting the subject’s desire for learning by gradually increasing the visual error [6].

General clinical stroke rehabilitation strategies are based on the motor learning theory. Motor learning is defined as “a set of processes associated with practice or experience that leads to relatively permanent changes in the ability to produce skilled action”. Feedback is known to be one of the key factors in achieving motor learning [27]. Stroke patients receive intrinsic or extrinsic feedback as a result of their task performance during rehabilitation. In particular, intrinsic feedback (e.g., sensory and visual feedback) allows for self-evaluations of performance to differentiate between incorrect and correct performance [24], in addition to allowing the error to be corrected directly when a problem occurs while performing a movement [28]. Therefore, accurate feedback on task performance is essential for the recovery of functional movement during stroke rehabilitation. However, in this study, error augmentation was applied, which hinders the accuracy of feedback by magnifying the error during the point-to-point movement of the upper limb. During the four weeks of upper-limb robot-assisted therapy based on visual error augmentation in virtual reality, the distance to the target on the monitor did not change, but the distance to the actual target increased gradually (0–15%). In other words, compared with the 1st week of training, the subjects had to move their arms 15% more in the 4th week of training to reach the target. As a result of these 4 weeks of training, the subjects showed improvement in the kinematic elements of point-to-point movements, as well as recovery of motor functions. Our findings corroborate those reported in previous studies, which showed improvement in upper-limb motor recovery as well as upper-limb movement patterns [7,29]. In addition, Liepert et al. reported that active training is more effective than passive training in improving motor performance and in promoting cerebral cortical reorganization [30]. Although the error augmentation strategy used in this study was contrary to the conventional concept of rehabilitation, we believe that the increase in visual error improved concentration on training and contributed to the improvement of active participation and motivation in training.

Another notable finding of this study was the lack of improvement in the upper-extremity distal part, as evidenced by the FMA distal unit after upper-limb robot-assisted therapy based on visual error augmentation in virtual reality. We believe that since the training provided in this study is simply a point-to-point movement that reaches the arm toward the targets, it did not improve the motor function of the distal part, including the wrist and hand. Most upper-limb robot training programs are designed to train specific movements, such as reaching out toward targets [31]. However, since many daily activities involve functional movements of the hand, and an advanced functional level of the upper-extremity distal part is required to lead a more independent daily life, future research should be performed on the error-augmentation paradigm that can improve the motor function of the upper-extremity distal part.

Our study has some limitations. First, given that this was a feasibility study, we did not have a control group with which these results could be compared. In the future, randomized, controlled trials should be performed to analyze the clinical effects of visual error augmentation in stroke survivors. Second, although most upper-limb movements in daily life are used in three dimensions, only simple, point-to-point movements were applied as training elements in this study. Thus, the development of more complex training protocols is needed to analyze the effect of training on activities performed in daily life. Third, because this study enrolled only chronic stroke survivors with high functional levels, the results may not be generalized to all patients. Finally, although NREH was created based on officially standardized sensors and encoders, and several usability tests were performed to evaluate the safety and reliability in healthy adults and stroke survivors before this feasibility study, future studies are needed on the intra- and inter-rater intervention reliability.

## 5. Conclusions 

This feasibility study was conducted to investigate the usefulness of visual error augmentation in virtual reality on upper-extremity motor recovery and kinematics in chronic stroke survivors. The results of this study showed that the upper-limb robot-assisted therapy based on visual error augmentation in virtual reality may have the potential to be used as one of the upper-limb rehabilitation strategies for chronic stroke survivors. In the future, additional RCT studies should be conducted to investigate the clinical effects of the virtual reality visual error-augmentation paradigm.

## Figures and Tables

**Table 1:** General characteristics and baseline clinical assessment of the subjects (*n* = 27).

Parameters	Mean ± SD or Number
Gender (male/female)	20/7
Paretic side (left/right)	13/14
Etiology (infarction/hemorrhage)	16/11
Brunnstrom stage (3/4/5/6)	1/17/8/1
MAS-UE (1/1+/2)	6/16/5
MRC-EF (3/4/5)	5/8/14
MRC-EE (3/4/5)	5/7/15
Age (years)	54.51 ± 12.44
Weight (kg)	67.88 ± 11.54
Height (cm)	167.62 ± 7.31
Onset duration (months)	12.70 ± 5.78
MMSE (scores)	26.70 ± 3.97
MBI (scores)	90.33 ± 6.25

MAS-UE: Modified Ashworth Scale-Upper Extremity, MRC: Medical Research Council, EF: elbow flexor, EE: elbow extensor, MMSE: Mini-Mental State Examination, MBI: Modified Barthel Index.

**Table 2:** Changes in upper-limb motor recovery according to upper-limb robot-assisted therapy based on visual error augmentation in virtual reality (*n* = 27).

Parameters		Pre-Intervention	Post-Intervention	∆ Change	Z Values	*p* Values
FMA	Total (max. 66)	36.92 ± 13.62	38.55 ± 13.94	1.62 ± 1.88	−3.562	<0.000
	Proximal (max. 42)	25.88 ± 7.74	27.11 ± 7.84	1.22 ± 1.64	−3.203	0.001
	Distal (max. 24)	11.03 ± 7.61	11.29 ± 7.76	0.25 ± 1.58	−0.962	0.336
BBT		5.33 ± 7.65	5.92 ± 8.16	0.59 ± 1.42	−2.032	0.042
ARAT (max. 57)		16.96 ± 17.70	18.29 ± 17.14	1.33 ± 1.54	−3.213	0.001

Values are expressed as mean ± SD. FMA-UE: Fugl–Meyer Assessment-Upper Extremity, Proximal: upper extremity and coordination/speed, Distal: wrist and hand, CS: coordination/speed, BBT: Box and Block Test, ARAT: Action Research Arm Test.

**Table 3:** Changes in upper-limb kinematics according to upper-limb robot-assisted therapy based on visual error augmentation in virtual reality (*n* = 27).

Parameters		Pre-Intervention	Post-Intervention	∆ Change	Z Value	*p* Value
Time (s)	IL	3.94 ± 1.60	2.94 ± 1.08	−0.99 ± 1.07	−4.036	<0.000
	Me	4.17 ± 1.70	2.93 ± 0.92	−1.24 ± 1.07	−4.325	<0.000
	CL	3.68 ± 1.43	3.00 ± 1.25	−0.68 ± 1.06	−3.099	0.002
Speed (cm/s)	IL	6.26 ± 3.80	8.28 ± 4.14	2.02 ± 3.60	−3.137	0.002
	Me	6.76 ± 4.02	8.93 ± 4.80	2.16 ± 3.95	−3.195	0.001
	CL	7.48 ± 5.55	9.98 ± 5.44	2.49 ± 4.15	−3.243	0.001
Distance (cm)	IL	15.09 ± 7.73	17.52 ± 7.27	2.42 ± 5.52	−3.555	<0.000
	Me	17.00 ± 8.06	19.59 ± 7.98	2.58 ± 5.66	−3.070	0.002
	CL	19.81 ± 11.47	22.46 ± 10.63	2.65 ± 6.85	−3.559	<0.000
CR	IL	0.61 ± 0.17	0.71 ± 0.13	0.09 ± 0.13	−2.246	0.025
	Me	0.61 ± 0.18	0.70 ± 0.13	0.08 ± 0.15	−1.970	0.049
	CL	0.61 ± 0.17	0.71 ± 0.12	0.09 ± 0.14	−2.499	0.012

Values are expressed as mean ± SD. $\mathrm{CR}:\;\mathrm{curvilinearity}\;\mathrm{ratio}=\frac{\mathrm{length}\;\mathrm{of}\;a\;\mathrm{straight}\;\mathrm{line}\;\mathrm{from}\;\mathrm{the}\;\mathrm{start}\;\mathrm{point}\;\mathrm{to}\;\mathrm{the}\;\mathrm{target}\;\mathrm{point}}{\mathrm{actual}\;\mathrm{displacement}\;\mathrm{of}\;\mathrm{the}\;\mathrm{hand}}$ IL: point-to-point movement toward ipsilateral, Me: point-to-point movement toward medial side, CL: point-to-point movement toward contralateral.

## Data Availability

The datasets used and analyzed during the current study are available from the corresponding author on reasonable request.
